# Exercise attenuates high-fat diet-induced PVAT dysfunction through improved inflammatory response and BMP4-regulated adipose tissue browning

**DOI:** 10.3389/fnut.2024.1393343

**Published:** 2024-05-09

**Authors:** Xiaojie Liu, Xi Jiang, Jing Hu, Mingxing Ding, Sang Ki Lee, Mallikarjuna Korivi, Yongdong Qian, Ting Li, Lifeng Wang, Wei Li

**Affiliations:** ^1^Exercise and Metabolism Research Center, College of Physical Education and Health Sciences, Zhejiang Normal University, Jinhua, China; ^2^School of Medicine, Jinhua Polytechnic, Jinhua, China; ^3^Department of Sport Science, College of Natural Science, Chungnam National University, Daejeon, Republic of Korea

**Keywords:** cardiovascular disease, high-fat diet, aerobic exercise, adipose tissue browning, BMP4

## Abstract

**Background:**

Perivascular adipose tissue (PVAT) dysfunction impairs vascular homeostasis. Impaired inflammation and bone morphogenetic protein-4 (BMP4) signaling are involved in thoracic PVAT dysfunction by regulating adipokine secretion and adipocyte phenotype transformation. We investigated whether aerobic exercise training could ameliorate high-fat diet (HFD)-induced PVAT dysfunction via improved inflammatory response and BMP4-mediated signaling pathways.

**Methods:**

Sprague-Dawley rats (*n* = 24) were divided into three groups, namely control, high-fat diet (HFD), and HFD plus exercise (HEx). After a 6-week intervention, PVAT functional efficiency and changes in inflammatory biomarkers (circulating concentrations in blood and mRNA expressions in thoracic PVAT) were assessed.

**Results:**

Chronic HFD feeding caused obesity and dyslipidemia in rats. HFD decreased the relaxation response of PVAT-containing vascular rings and impaired PVAT-regulated vasodilatation. However, exercise training effectively reversed these diet-induced pathological changes to PVAT. This was accompanied by significantly (*p* < 0.05) restoring the morphological structure and the decreased lipid droplet size in PVAT. Furthermore, HFD-induced impaired inflammatory response (both in circulation and PVAT) was notably ameliorated by exercise training (*p* < 0.05). Specifically, exercise training substantially reversed HFD-induced WAT-like characteristics to BAT-like characteristics as evidenced by increased UCP1 and decreased FABP4 protein levels in PVAT against HFD. Exercise training promoted transcriptional activation of BMP4 and associated signaling molecules (p38/MAPK, ATF2, PGC1α, and Smad5) that are involved in browning of adipose tissue. In conjunction with gene expressions, exercise training increased BMP4 protein content and activated downstream cascades, represented by upregulated p38/MAPK and PGC1α proteins in PVAT.

**Conclusion:**

Regular exercise training can reverse HFD-induced obesity, dyslipidemia, and thoracic PVAT dysfunction in rats. The browning of adipose tissue through exercise appears to be modulated through improved inflammatory response and/or BMP4-mediated signaling cascades in obese rats.

## 1 Introduction

Cardiovascular diseases (CVDs) are the leading cause of death worldwide, accounting for ~18 million deaths annually (1). CVDs are a group of disorders characterized by the disruption of vascular homeostasis, which is emerging as a key instigator in disease pathogenesis (2, 3). Perivascular adipose tissue (PVAT), a unique tissue around the blood vessel, provides mechanical support to blood vessels and is involved in all aspects of vascular physiology and pathophysiology (4). PVAT plays a critical role in vascular homeostasis by secreting the inflammatory cytokines, hormones, and growth factors (5, 6). Depending on its existing place, PVAT shows phenotypic, genotypic, and functional heterogeneity. Particularly, PVATs around the thoracic aorta exhibit brown adipose tissue (BAT)-like characteristics (7), and any alterations in the adipocyte phenotype leads to PVAT dysfunction. Studies have confirmed that chronic high-fat diet or obesity increases the PVAT mass and develops a white adipose tissue (WAT)-like phenotype (8, 9). Whitening of PVAT during obesity represents excessive production of free radicals and subsequent occurrence of oxidative stress. In contrast to BAT-like characteristics, whitening of PVAT shows larger lipid droplets, lower expression of uncoupling protein-1 (UCP-1), and low mitochondrial number (10). This phenomenon is accompanied by an increased inflammatory response, which decreases the vascular relaxation response, regulated by PVAT (11).

PVAT dysfunction and associated cardiovascular complications can be prevented by targeting the restoration of BAT-like characteristics. The acquisition of an adipocyte phenotype involves the differentiation of adipose stem and progenitor cells (ASPCs) and the direct transformation of the adipocyte phenotype. Lee et al. demonstrated that platelet-derived growth factor receptor α (PDGFRα) cells, a type of ASPCs, can differentiate into white or brown adipocytes *in vivo* depending on the type of inductive signal (12). In addition, the acquisition of brown adipocytes in the white adipose depot after cold stimulation was derived from the direct transformation of white adipocytes (13). Meanwhile, bone morphogenetic protein 4 (BMP4) is closely associated with adipogenesis and remodeling (14). At the molecular level, overexpression of BMP4 in adipocytes has been reported to activate downstream Smads and p38/MAPK signaling pathways, and such activation promotes the browning of WAT and impedes metabolic disturbances induced by a high-fat diet (15–17). Evidence has shown that the absence of BMP4 leads to impaired self-metabolism in PVAT. Conversely, overexpression of BMP4 stimulates the browning of PVAT, inhibits inflammatory responses, and hampers the development of atherosclerosis (18). Furthermore, knockdown of BMP4 in PVAT was found to accelerate obesity or angiotensin II (Ang II)-induced hypertension and vascular remodeling (19). Overall, these findings demonstrate that BMP4 is involved in the regulation of PVAT metabolism and protects the cardiovascular system.

It is well-documented that sedentary habits and/or excessive consumption of fat-enriched foods over a period of time trigger the onset of PVAT dysfunction by promoting the production of inflammatory cytokines and reactive oxygen species (ROS) (20, 21). On the other hand, regular exercise training plays a crucial role in prevention of diet-induced cardiovascular and other metabolic diseases (22, 23). Particularly, aerobic exercise can promote the browning of PVAT and alleviates high-fat diet-induced PVAT dysfunction, possibly by reducing inflammation and oxidative stress in obese mice (24). A recent study has shown that aerobic exercise training ameliorates high-fat diet-induced PVAT dysfunction (25); however, a precise mechanism remains unclaimed. Moderate treadmill exercise has been reported to increase the number of brown adipocytes and UCP-1 expression in PVAT of diabetic mice (26). Although this occurrence is considered to be associated with attenuation of oxidative stress or inflammation, the detailed molecular mechanism was not elucidated. In addition, the effect of aerobic exercise on high-fat diet-induced PVAT phenotypic shifts through the regulation of BMP4 remains unclear. Therefore, this study aimed to investigate the effect of aerobic exercise training against high-fat diet-induced PVAT dysfunction and to explore the molecular mechanism involved in BMP4-regulated adipose tissue browning in an obese rat model.

## 2 Materials and methods

### 2.1 Chemicals and reagents

Triglyceride kit (Cat. A110-1-1), total cholesterol kit (Cat. A111-1-1), low-density lipoprotein cholesterol kit (Cat. A113-1-1), and high-density lipoprotein cholesterol kit (Cat. A112-1-1) were purchased from Nanjing Jiancheng Bioengineering (Nanjing, China). Leptin ELISA kit (Cat. H147-1-2), adiponectin ELISA kit (Cat. H179-1-2), interleukin-1β ELISA kit (Cat. H002-1-2), and tumor necrosis factor-alpha ELISA kit (CAS#. H052-1-2) were purchased from Sangon Biotech (Shanghai, China). NaCl (CAS#. 7647-14-5), KCl (CAS#. 7447-40-7), CaCl_2_ (CAS#. 10043-52-4), K_2_HPO_4_ (CAS#. 16788-57-1), NaHCO_3_ (CAS#. 144-55-8), glucose (CAS#. 14431-43-7) were purchased from HUSHI (Shanghai, China). L-phenylephrine hydrochloride (CAS#. 59-42-7) and acetylcholine (CAS#. 60-31-1) were purchased from Shanghai Aladdin Biochemical Technology (Shanghai, China). RIPA lysis buffer, protease, phosphatase inhibitor (Cat. A32959), Rapid Gold BCA Protein Assay Kit (Cat. A53226), and enhanced chemiluminescence solution (Cat. 34578) were purchased from Thermo Fisher Scientific (Avebue Waltham, MA, USA). Polyvinylidene difluoride membranes (Cat. 1,620,177) and skimmed milk (Cat. 1,706,404) were purchased from Bio-Rad (CA, USA). Primary antibodies against BMP4 (12492-1-AP), PGC1α (66369–1-Ig), UCP1 (23673-1-AP), FABP4 (12802-1-AP), and GAPDH (60004-1-Ig) were purchased from the Proteintech group (Wuhan, China). The primary antibodies against Phosphorylated-p38/MAPK (9211S) and p38/MAPK (8690S) were purchased from Cell Signaling Technology (MA, USA). The Proteintech group (Wuhan, China) provided goat anti-mouse (SA00001–1) while Cell Signaling Technology (MA, USA) supplied goat anti-rabbit (7,074) as the secondary antibodies. Fluorescently labeled primary antibodies against UCP-1 (23673-1-AP) and FABP4 (12802-1-AP) were purchased from the Proteintech group (Wuhan, China). Then, the PDGFRα (AB 203491) and Ki67 (AB 16667) were purchased from Abcam (Cambridge, UK). Fluorescently labeled secondary antibodies against Goat pAB to Rb IgG (AB 150080) was purchased from Abcam (Cambridge, UK). Antifade with DAPI (cat. P0131) was purchased from Beyotime Biotechnology (Shanghai, China). Trizol was purchased from Thermo Fisher Scientific (Avebue Waltham, MA, USA). PrimeScript RT Master Mix (Perfect Real Time) Kit (Cat. RR036A) was purchased from Takara Bio (Shiga, Japan). PowerUpTM SYBRTM Green Master Mix (Cat. A25742) was purchased from Thermo Fisher Scientific (Avebue Waltham, MA, USA). Gene-specific primers were purchased from Sangon Biotech (Shanghai, China).

### 2.2 Animal care and grouping

Six-week-old male Sprague-Dawley (SD) rats (*n* = 24), weighing 257 ± 10 gm, were purchased from the Vital River (Zhejiang, China). The rats were kept in a specific pathogen-free chamber with a 12 h light and 12 h dark cycle. The room temperature was maintained at 23 ± 2°C with a humidity of 50–60%. All rats had free access to water and food. After a 1-week adaptation period to the laboratory conditions, rats were randomly assigned into three groups, namely control (Ctrl, *n* = 8), high-fat diet (HFD, *n* = 8), and high-fat diet plus exercise training (HEx, *n* = 8). Rats in the control group received a normal diet (Research Diets, New Brunswick, NJ, USA), while those in the HFD and HEx groups fed a high-fat diet containing 60% of fat (Research Diets, New Brunswick, NJ, USA) for a duration of 6 weeks. Along with high-fat diets, rats in the HEx group performed aerobic exercise training in accordance with the exercise protocol. The entire study design and protocols were reviewed and approved by the Zhejiang Normal University Animal Ethical Committee with the reference number ZSDW2022027.

### 2.3 Exercise protocol

In the beginning, rats in the HEx group were trained on a motorized rodent treadmill to familiarize the treadmill running (ZH-PT, Anhui Zhenghua Biologic Apparatus Facilities, Anhui, China). During acclimation, the treadmill speed was set at 16 m/min for 20 min on day 1. Then, the speed was gradually increased by 2 m/min, and the time was increased by 10 min until reaching the target speed of 24 m/min and time of 60 min. The final exercise program consisted of 60 min/day, 5 days/week at a 0° incline for a period of 6-week. The exercise intensity corresponded to a maximum oxygen (O_2_) uptake of 60–70% (27). The exercise program was scheduled between 5:00 and 7:00 p.m. from Monday to Friday.

### 2.4 Sample collection and biochemical assays

The rats were anesthetized (urethane 2 g/kg, intraperitoneal injection) 48 h after the last exercise training session, followed by 12 h fasting. The PVAT samples were extracted and weighed using a laboratory balance (Srtorius Balances, Gottingen, Germany). For PCR analysis, the PVATs were rinsed in ice-cold saline and were rapidly frozen in liquid nitrogen. The tissues were then stored at −80°C using a deep freezer (Thermo Fisher Scientific, Waltham, USA). The PVATs designated for staining were embedded in an optimal cutting temperature compound (Sakura, Tokyo, Japan) and frozen in isopentane (Macklin, C14950843, Shanghai, China). They were then either stored at −80°C or used immediately. The blood samples (5 ml) were collected into an anticoagulant tube via venipuncture. The blood samples were centrifuged at 3,000 rpm for 15 min. Sera were prepared and used for measurements. Total triglycerides (TG), total cholesterol (TC), low-density lipoprotein-cholesterol (LDL-C), and high-density lipoprotein-cholesterol (HDL-C) levels were determined using the Beckman Coulter AU680 automatic biochemical analysis device (Beckman Counter, Brea, USA). The changes in leptin, adiponectin, interleukin-1β (IL-1β), and tumor necrosis factor-alpha (TNF-α) were determined by the commercially available ELISA kits (Sangon Biotech, Shanghai, China).

### 2.5 Vascular ring experiment

In a tray with phosphate buffered saline, the thoracic aorta containing PVAT was sliced into vascular rings measuring 2–3 mm, and these rings were then fixed in different tension transducers (ADInstruments, Bella Vista, NSW, Australia) in a perfusion tank kept at a constant temperature of 37°C, with continuous ventilation comprising 5% of CO_2_ and 95% of O_2_. Kreb's buffer solution (99.01 mM of NaCl, 4.69 mM of KCl, 1.89 mM of CaCl_2_, 1.2 mM of MgSO_4_, 1.03 of mM K_2_HPO_4_, 25 mM of NaHCO_3_, and 11.1 mM of glucose at a pH level of 7.4) was added, and the response of each rat vessel was observed. Then, 60 mM of KCl, 0.3 μM of L-phenylephrine hydrochloride (PE), and various concentrations (10^−6^-10^−2^ M) of acetylcholine (ACh) were consecutively added to determine the vascular reactivity, and real-time data were recorded, analyzed, and archived using LabChart software (ADInstruments, Bella Vista, NSW, Australia) (28).

### 2.6 Assessment of lipid droplets by Hematoxylin and Eosin (H&E) staining

After being fixed with an optimal cutting temperature compound, the thoracic aortic segments with PVAT were cut into small sections of 10 μm thickness, and the H&E staining was performed according to a standard protocol (29). The lipid droplet area was calculated using ImageJ software (National Institutes of Health, USA), and the average area was calculated based on 10 complete lipid droplets in one field of view.

### 2.7 Assessment of protein fluorescence intensity by immunofluorescence staining

Sections of the thoracic aortic PVAT were prepared and incubated with a primary antibody mixture of UCP1 (dilution: 1:100, Proteintech, Wuhan, China), FABP4 (dilution: 1:100, Proteintech, Wuhan, China), PDGFRα (dilution: 1: 100, Abcam, Cambridge, UK), or Ki67 (dilution: 1:100, Abcam, Cambridge, UK) at 4°C overnight. The samples were protected from light exposure, and Goat pAb to Rb IgG (dilution: 1:500, Abcam, Cambridge, UK) secondary antibody mixture was added and incubated for 1 h at room temperature before sealing with a DPAI-containing sealer (Beyotime, Shanghai, China). Finally, the stained tissues were examined under a fluorescence microscope (Leica, Weztlar, Germany). The fluorescence index was determined using Image J software (National Institutes of Health, USA).

### 2.8 Western blot

Total proteins were extracted from rat thoracic aorta PVAT using RIPA lysis buffer supplemented with a protease and phosphatase inhibitor mixture (Thermo Fisher Scientific, Waltham, USA). The total protein content was measured using the Rapid Gold BCA Protein Assay Kit (Thermo Fisher Scientific, Waltham, USA). Protein (30 μg) was separated by electrophoresis on sodium dodecyl sulfate (SDS)-polyacrylamide gels ranging from 10 to 12%. The separated proteins were transferred onto polyvinylidene difluoride membranes (Bio-Rad, CA, USA) and were then blocked with 5% skimmed milk (Bio-Rad, CA, USA) for 1 h. Following this, the membranes were washed three times with TBST buffer and incubated overnight at 4°C with respective primary antibodies, such as BMP4, UCP1, FABP4, PGC1α, GAPDH (Proteintech, Wuhan, China), P-p38/MAPK, and p38/MAPK (Cell Signaling Technology, MA, USA). After TBST washing, membranes were probed with secondary antibodies (goat anti-mouse from Proteintech and goat anti-rabbit from Cell Signaling Technology) for 2 h at room temperature. Following three additional TBST washes, we applied an enhanced chemiluminescence solution (Thermo Fisher Scientific, Waltham, USA), and the membranes were visualized using a chemiluminescence imaging system (Bio-Rad, CA, USA). The intensity of the bands was quantified using ImageJ software (National Institutes of Health, USA).

### 2.9 Quantitative real-time polymerase chain reaction

The PVAT was lysed using Trizol lysis solution, followed by the extraction of the total RNA, and the obtained total RNA was reverse transcribed into cDNA using PrimeScript RT Master Mix (Perfect Real Time) (Takara, Shiga, Japan). A qPCR assay was carried out using PowerUpTM SYBRTM Green Master Mix (Thermo Fisher Scientific, Waltham, USA). The reaction conditions were pre-denaturation at 95°C for 5 min, denaturation at 95°C for 10 s, and annealing at 60°C for 30 s, 40 cycles. Gene expression levels were calculated using the 2^−Δ*ΔCt*^ method with PPIA as an internal reference. PPIA is reported as a more stable housekeeping marker in diet-induced obese animal models (30). The primer sequences are listed in Table 1.

**Table 1:** Primers for qPCR used in this study.

**Rat gene**	**Forward**	**Reverse**
*Ppia*	AGGATTCATGTGCCAGGGTG	CTCAGTCTTGGCAGTGCAGA
*Bmp4*	CGGACCACCTCAACTCAACCAATC	CAACACCACCTTGTCGTACTCGTC
*p38/MAPK*	ACAGTCCTCTCCTCTCCTCTCCTC	TCGGTTCTCCCTTTGTTCGGTTTG
*Pgc1α*	GCCACTACAGACACCGCACAC	GTATTCGTCCCTCTTGAGCCTTTCG
*Smad1*	GAAGAAGAGAAATGGGCGGAGAAGG	GAGCGAGGAATGGTGACACAGTTAC
*Smad5*	GAGAACACCAGGCGGCACATC	CACTAAGACACTCGGCATACACCTC
*Smad8*	GCTTGTTCAAGGACCTGCGAGAG	GCTGCTTGCTTGCTGGATGTTTC
*Leptin*	AAAGAGTCCCAAGTGCCACAGTC	ATGAGGTGACCAAGGTGACATAGC
*Adiponectin*	GATGGCAGAGATGGCACTCCTG	CCCTTCCGCTCCTGTCATTCC
*Mcp-1*	AGCCCAGAAACCAGCCAACTC	GCCCAGAAGCGTGACAGAGAC
*Il-10*	AAGGCAGTGGAGCAGGTGAAG	TGAGTGTCACGTAGGCTTCTATGC
*Atf2*	CCACATCAGCTATCGTTCGTCCAG	AGGTTGGTGAAGGTACTGCTTGTTG

### 2.10 Statistical analysis

All data were presented as mean ± standard error of the mean (SEM). Statistical Package for the Social Sciences (SPSS) software version 25.0 (IBM, Chicago, USA) was employed to calculate the descriptive statistics. The one-way Analysis of Variance (ANOVA) was employed to determine the significance of differences, followed by Bonferroni *post-hoc* tests. The differences were considered statistically significant at *p* < 0.05.

## 3 Results

### 3.1 Aerobic exercise training diminishes HFD-induced body weight gain

We found that high-fat diet feeding to rats for 6 weeks significantly (*p* < 0.05) increased body weight compared to the normal diet. The weight gain from HFD was noticed starting in week 2, became statistically significant by week 3, and continued until week 6 compared to the control group. Rats with a notable weight gain (~10%) with HFD in a period of 6 weeks were considered obese rats (31). However, aerobic exercise training significantly (*p* < 0.05) diminished the HFD-induced weight gain from week 3 and maintained significantly lower body weights until week 6 (Supplementary Figure S1).

### 3.2 Aerobic exercise decreases serum lipid profiles in HFD-induced obese rats

As shown in Table 2, circulating TC, TG, and LDL-C levels were significantly (*p* < 0.05) higher in the HFD group than that in the control group. A paramount reduction of TC, TG, and LDL-C levels were observed against the high-fat diet after 6 weeks of aerobic exercise (*p* < 0.05). The decreased lipid profiles with exercise in obese rats were almost similar to the control values. Nevertheless, the HDL-C levels remained unchanged with exercise or high-fat diet in this study (Table 2).

**Table 2:** Lipid profile and inflammatory biomarkers in control (Ctrl), high-fat diet (HFD), and high-fat diet plus exercise (HEx) groups.

**Biomarkers (units)**	**Groups**		
	**Ctrl**	**HFD**	**HEx**
TC (mg/dl)	26.2 ± 2.3 (6)	41.5 ± 4.7^*^ (6)	28.5 ± 1.2^#^ (6)
TG (mg/dl)	19.2 ± 1.3 (6)	28.0 ± 2.3^*^ (6)	17.4 ± 2.5^#^ (6)
LDL-C (mg/dl)	8.4 ± 0.8 (6)	14.1 ± 1.7^*^ (6)	9.5 ± 0.4^#^ (6)
HDL-C (mg/dl)	17.4 ± 0.7 (6)	17.7 ± 1.9 (6)	17.6 ± 0.7 (6)
Leptin (ng/dl)	2.0 ± 0.2 (7)	7.4 ± 0.5^*^ (6)	4.1 ± 0.2^#^ (6)
Adiponectin (ng/dl)	9.0 ± 1.0 (7)	2.5 ± 0.5^*^ (8)	4.0 ± 0.6^*^ (7)
IL-1β (pg/dl)	112.5 ± 3.4 (6)	124.9 ± 3.6 (8)	102.7 ± 6.2^#^ (6)
TNF-α (pg/dl)	77.6 ± 7.9 (7)	78.2 ± 6.7 (7)	73.3 ± 11.0 (6)

The data are presented as mean ± SEM. The number of animals per group is indicated in the parentheses. The results are significant compared to the control group (^*^p < 0.05) and the HFD group (^#^*p* < 0.05).

### 3.3 Exercise attenuates HFD-induced inflammatory response

The concentrations of pro- and anti-inflammatory cytokines in the serum of obese rats were assessed to determine the effects of aerobic exercise against HFD-induced inflammation. Compared with the control group, the HFD group exhibited enormously increased serum leptin levels and substantially decreased adiponectin concentrations. However, this response was partially reversed by exercise training in the HEx group (Table 2). We further noticed that HFD-induced increased tendency in IL-1β concentrations was significantly decreased by exercise training (Table 2). The changes in TNF-α levels were not significantly different between high-fat diet and exercise groups (Table 2).

### 3.4 Exercise training promotes PVAT-regulated vasodilatation against HFD

**Figure 1:**
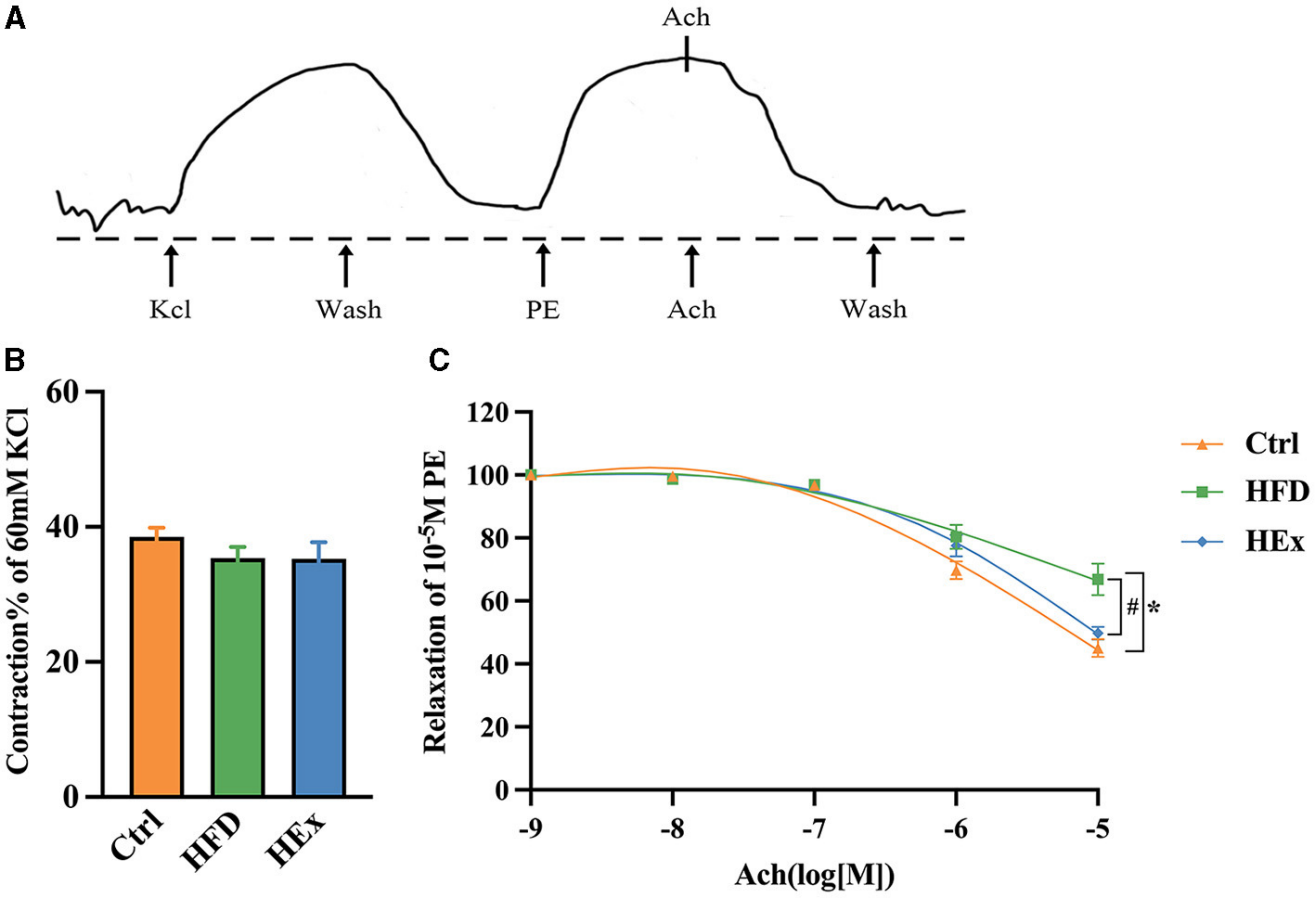
The effects of aerobic exercise on vasoconstriction and vasodilation in rats fed with a high-fat diet. **(A)** Representative trace showing aortic ring reaction in response to different treatments. **(B)** Quantitative analysis of systolic tension in aortic rings containing PVAT (*n* = 6–7). **(C)** ACh-induced vasodilatory response in aortic rings containing PVAT (*n* = 6–7). The data are presented as mean ± SEM. The results are significant compared to the control group (*p < 0.05) and the HFD group (^#^*p* < 0.05).

Changes in vascular reactivity in response to HFD and HFD combined with exercise were determined by vascular ring experiments. The aortic ring reactions in response to potassium chloride (KCl), phenylephrine (PE), and acetylcholine (ACh) treatments were presented in Figure 1A. The results showed that the maximal contractile response of isolated thoracic aorta to KCl (60 mM) was not significantly different among the groups (Figure 1B). We found that PVAT-containing aortic rings in HFD rats exhibited a significantly (*p* < 0.05) reduced relaxation response to ACh (10^−2^ M) induction. This observation is further supported by increased fat accumulation. However, the relaxation response to ACh was significantly (*p* < 0.05) restored when HFD combined with exercise training (HEx), as depicted in Figure 1C. These findings suggest that 6-week aerobic exercise can reverse the high-fat diet-induced PVAT dysfunction (contractile), thereby protecting vascular function in obese rats.

### 3.5 Exercise reverses HFD-induced histomorphometric changes and lipid droplet deposition in PVAT

**Figure 2:**
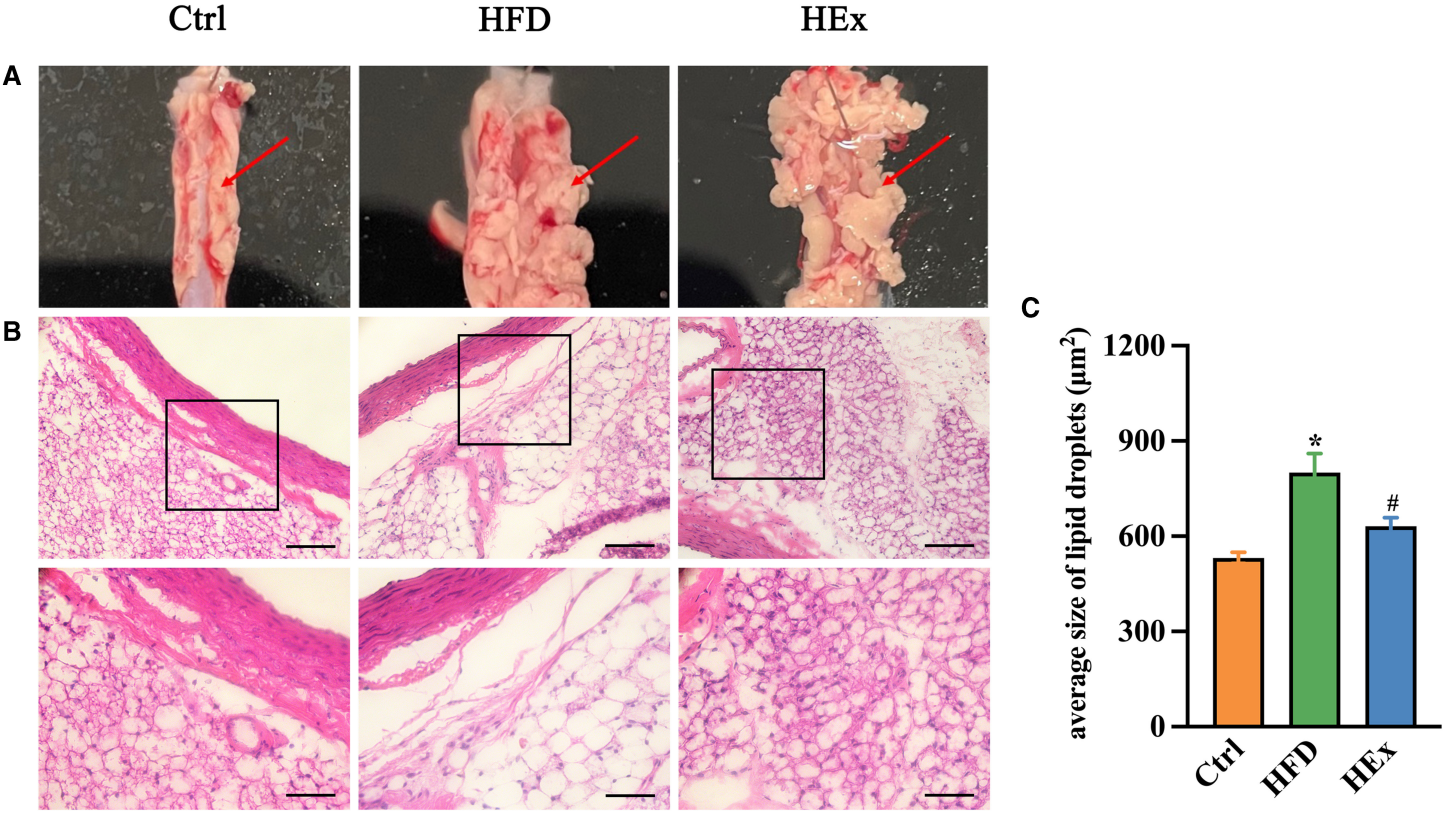
Analysis of the characteristics of PVAT. **(A)** Morphology of PVAT in thoracic aortic segments of rats. **(B)** Representative images of H&E staining of aorta PVAT (*n* = 6). Digital images were captured using 20X and 40X magnification (middle and lower panels). Scale bar = 100/50 μm. **(C)** Quantified data showing the average size of lipid droplets in PVAT (*n* = 6). The data are presented as mean ± SEM. The results are significant compared to the control group (*p < 0.05) and the HFD group (^#^*p* < 0.05).

The effects of high-fat diet and aerobic exercise on PVAT morphological changes and WAT/BAT-like characteristics were assessed through histopathological studies and presented in Figure 2. Images from the control group evidenced normal morphology of thoracic aorta PVAT. However, obese rats in the HFD group exhibited a greatly increased volume of PVAT and morphological derangement (Figures 2A, B). Interestingly, exercise training reversed the HFD-induced tissue enlargement and structural derangements as shown in Figures 2A, B. The quantified area of lipid droplets in PVAT of HFD rats was significantly (*p* < 0.05) greater compared to the control group. This indicates a WAT-like characteristic, suggesting profusely altered adipose tissue architectural stature. It is worthy to note that exercise training substantially (*p* < 0.05) reduced the size of lipid droplet area in PVAT, implying that exercise can restore the PVAT morphology in obese rats (Figures 2B, C).

### 3.6 Effect of exercise on phenotypic transformation of PVAT against HFD

**Figure 3:**
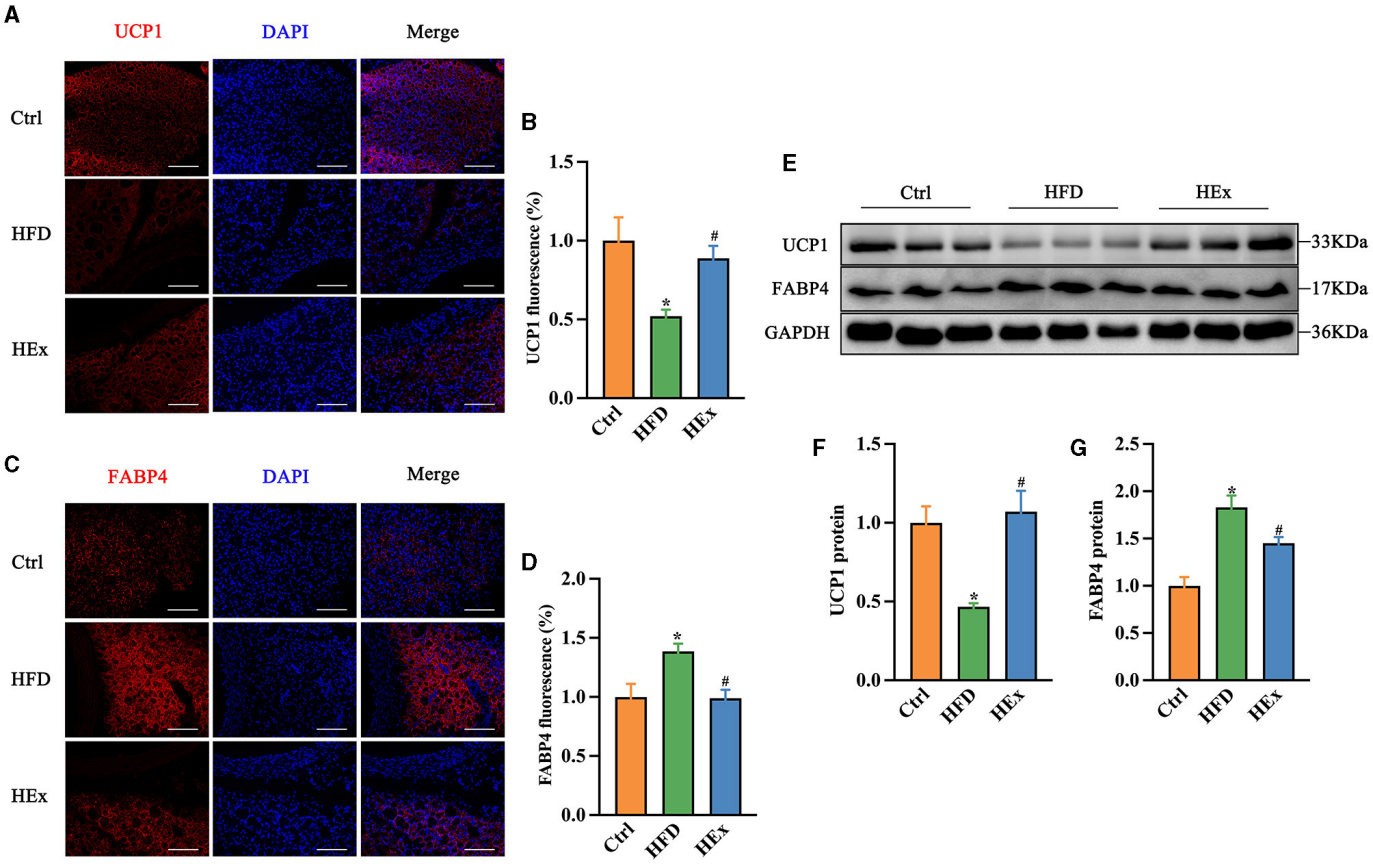
Exercise alters the brown and white adipose tissue markers in PVAT of rats fed a high-fat diet. **(A, C)** Representative images showing the positive staining for UCP1 and FABP4. Scale bar: 100 μm. Red: UCP1 or FABP4; blue: nuclei counterstained with DAPI. **(B)** UCP1 fluorescence was quantified and presented as histogram (*n* = 7–8). **(D)** FABP4 fluorescence was quantified and presented as histogram (*n* = 6). **(E)** Representative Western blot images of UCP1, FABP4, and internal control GAPDH in PVAT. **(F, G)** Protein levels of UCP1 and FABP4 (*n* = 6). The data are presented as mean ± SEM. The results are significant compared to the control group (*p < 0.05) and the HFD group (^#^*p* < 0.05).

Immunofluorescence and Western blot analyses were performed to assess the adipose tissue phenotypic transformation in response to HFD and HEx treatments. For this, the expression of brown and white adipose tissue markers, UCP1 and fatty acid binding protein 4 (FABP4), was determined in PVAT. Immunofluorescence images showed that the UCP1 expression was significantly reduced (Figures 3A, B), and the FABP4 expression was substantially enhanced (Figures 3C, D) with HFD compared to the control diet. These findings witnessed the whitening of PVAT from chronic HFD feeding. However, exercise training considerably increased UCP1 expression (Figures 3A, B) and reduced FABP4 expression in PVAT against HFD (Figures 3C, D).

Then, we continued with Western blot assays to further examine the proteins levels of UCP1 and FABP4 in PVAT. Similar to immunofluorescence evidence, we found that HFD suppressed UCP1 while enhancing FABP4 protein levels in adipose tissue. This HFD-induced whitening phenomenon was effectively reversed by exercise training, as shown by a significant restoration of UCP1 and inhibition of FABP4 proteins levels (Figures 3E–G). The restored UCP1 amount with exercise training is approximately 2-fold higher than HFD-induced loss (Figures 3E, F). Collectively, these observations demonstrate that 6-week aerobic exercise can reverse the HFD-induced WAT-like changes in thoracic PVAT.

### 3.7 Exercise ameliorates HFD-induced inflammatory response in PVAT

**Figure 4:**
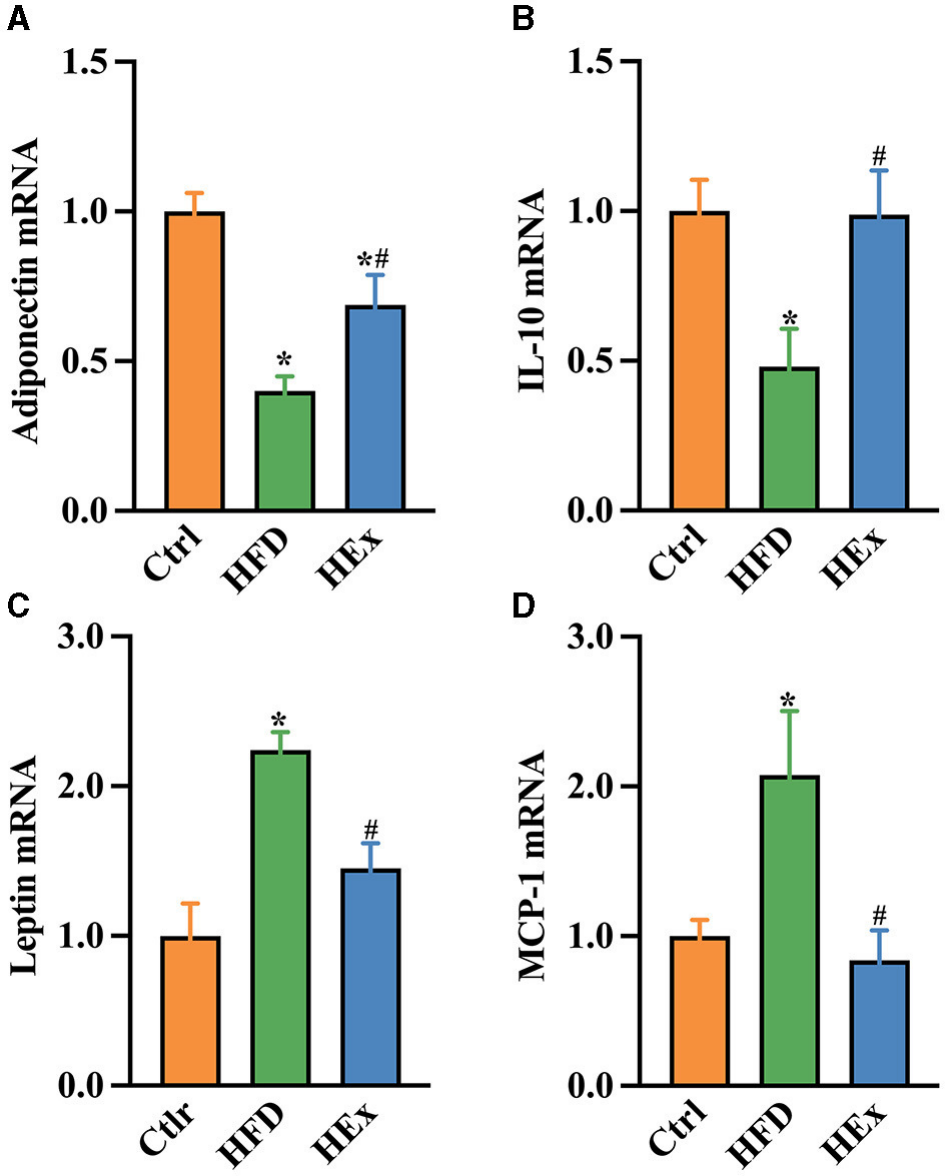
Gene expressions of pro- and anti-inflammatory biomarkers in PVAT of rats with HFD and HEx treatments. Relative changes in mRNA levels of adiponectin **(A)**, IL-10 **(B)**, leptin **(C)**, and MCP-1 **(D)** in PVAT of rats (*n* = 6). The data are presented as mean ± SEM. The results are significant compared to the control group (*p < 0.05) and the HFD group (^#^*p* < 0.05).

Changes in the mRNA expressions of pro- and anti-inflammatory cytokines were determined to address the influence of exercise on the inflammatory response in PVAT of obese rats. We found that HFD significantly decreased adiponectin and IL-10 mRNA expressions in PVAT. The decreased mRNA expressions of both adiponectin and IL-10 with HFD were more than 50% (Figures 4A, B). Furthermore, HFD triggered the PVAT-derived pro-inflammatory response as evidenced by elevated leptin and monocytes chemoattractant protein-1 (MCP-1) mRNA expressions (Figures 4C, D). These results emphasize the impaired tissue inflammatory system following HFD. Nevertheless, exercise training combined with HFD not only restored (*p* < 0.05) the adiponectin and IL-10 mRNA levels (Figures 4A, B) but also attenuated (*p* < 0.05) the leptin and MCP-1 mRNA levels in PVAT (Figures 4C, D). Overall, our findings revealed that aerobic exercise is effective in suppressing the HFD-induced inflammatory response in thoracic PVAT.

### 3.8 Effect of exercise on proliferation of ASPCs in PVAT of obese rats

**Figure 5:**
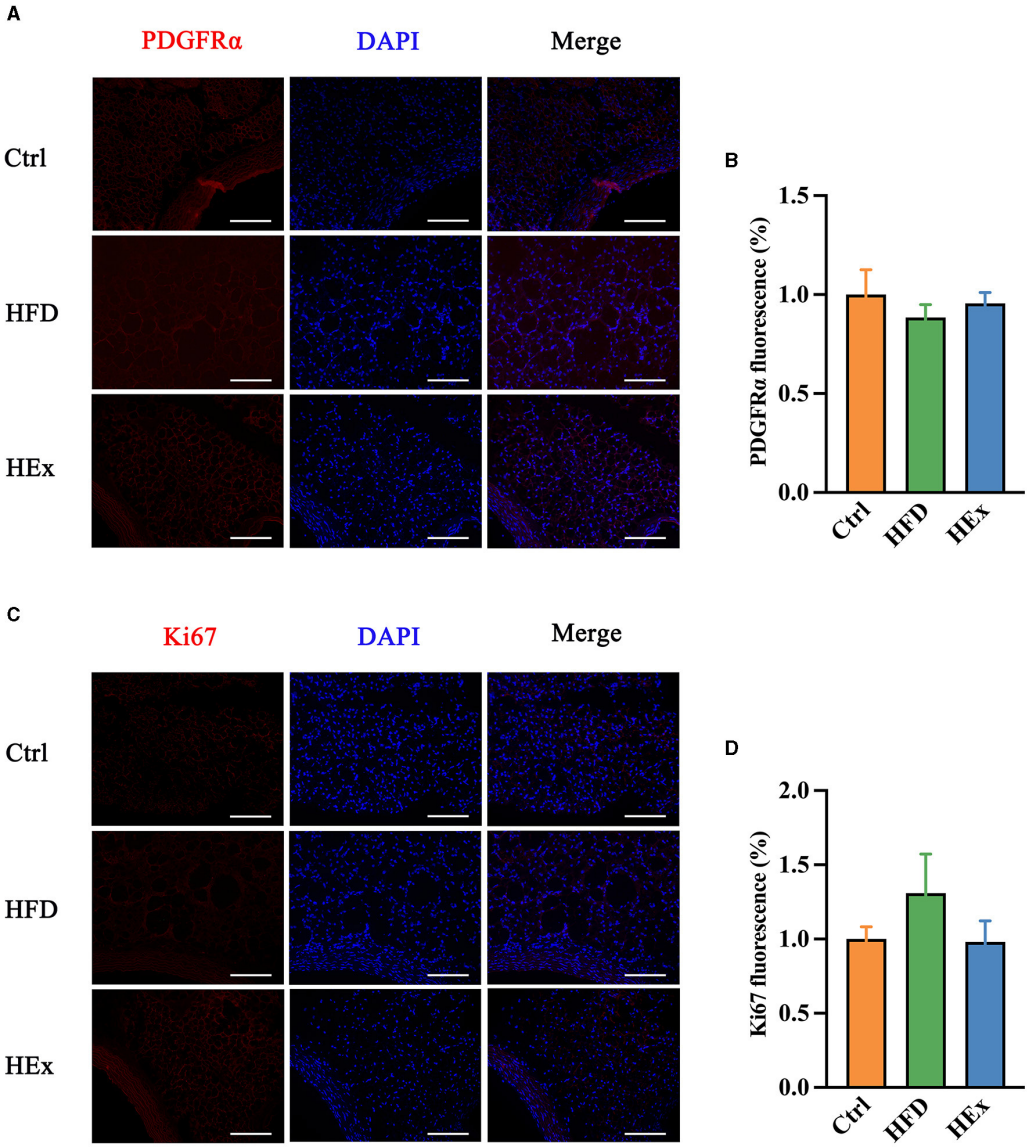
Effects of aerobic exercise on proliferation of ASPCs in PVAT. **(A, C)** Representative images showing positive staining for PDGFRα and Ki67. Scale bar: 100 μm. Red: PDGFRα or Ki67; blue: nuclei counterstained with DAPI. **(B)** PDGFRα fluorescence was quantified and presented as a histogram (*n* = 6–7). **(D)** Ki67 fluorescence was quantified and presented as a histogram (*n* = 6). The data are presented as mean ± SEM.

Identifying factors that trigger the adipose tissue phenotypic transformation is an important aspect to explain the reasons for PVAT dysfunction in obese animal models. Therefore, we tested the changes in PDGFRα and Ki67 proteins to demonstrate ASPC proliferation in response to HFD and exercise intervention. Images from immunofluorescence studies showed that either HFD or aerobic exercise had no significant impact on proliferation of ASPCs in PVAT. The fluorescent intensity of PDGFRα in HFD and HEx groups was not different from the control (Figures 5A, B). The Ki67 appears to be overexpressed with HFD, but the quantified fluorescence intensity was not statistically different compared with the control (Figures 5C, D). Therefore, it is presumed that phenotypic transformation in PVAT of obese rats may be mediated by other complex alterations in adipocyte metabolism, which should be further investigated.

### 3.9 Exercise modulates BMP4 signaling pathway in PVAT of obese rats

**Figure 6:**
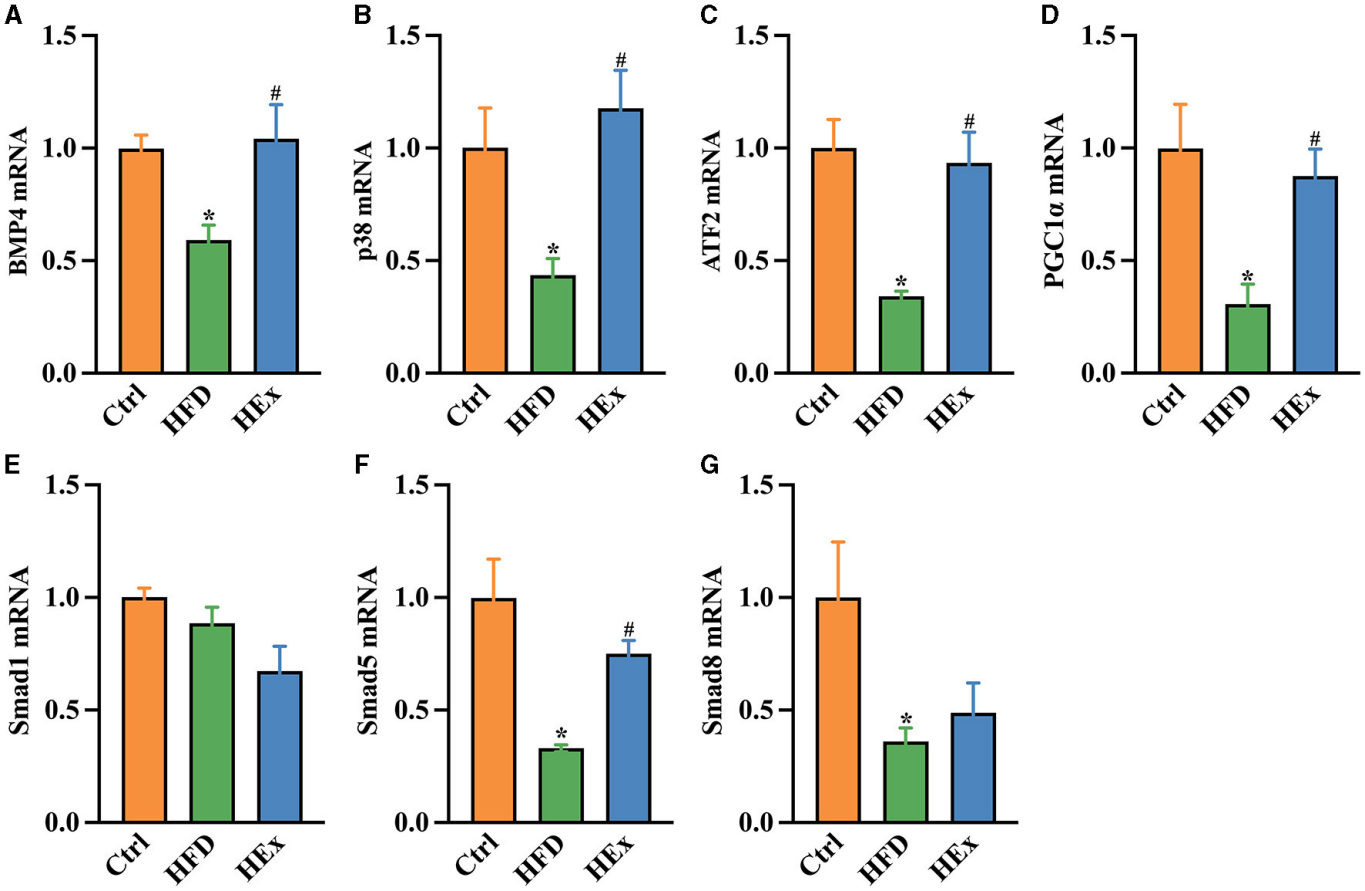
Effects of aerobic exercise on the BMP4 signaling pathway in PVAT of rats fed with a high-fat diet. Relative mRNA levels of BMP4 **(A)**, p38 **(B)**, ATF2 **(C)**, PGC1β **(D)**, Smad1 **(E)**, Smad5 **(F)**, and Smad8 **(G)** in PVAT of rats (*n* = 6). The data are presented as mean ± SEM. The results are significant compared to the control group (*p < 0.05) and the HFD group (^#^*p* < 0.05).

To further underscore whether PVAT phenotype transformation is regulated by BMP4, we examined the transcriptional activation and protein levels of key molecules that are involved in BMP4 signaling pathways. The qPCR results showed that the mRNA expressions of BMP4 and associated molecules, including p38/MAPK, ATF2 (activating transcription factor 2), PGC1α (peroxisome proliferator-activated receptor gamma coactivator 1α), Smad5, and Smad8 were substantially (*p* < 0.05) downregulated with HFD compared with the control diet (Figures 6A–D, F, G). Conversely, the mRNA expressions of BMP4, p38/MAPK, ATF2, PGC1α, and Smad5 were significantly (*p* < 0.05) upregulated when HFD combined with exercise (Figures 6A–D, F). Nevertheless, no significant change in Smad1 was observed among the groups (Figure 6E).

**Figure 7:**
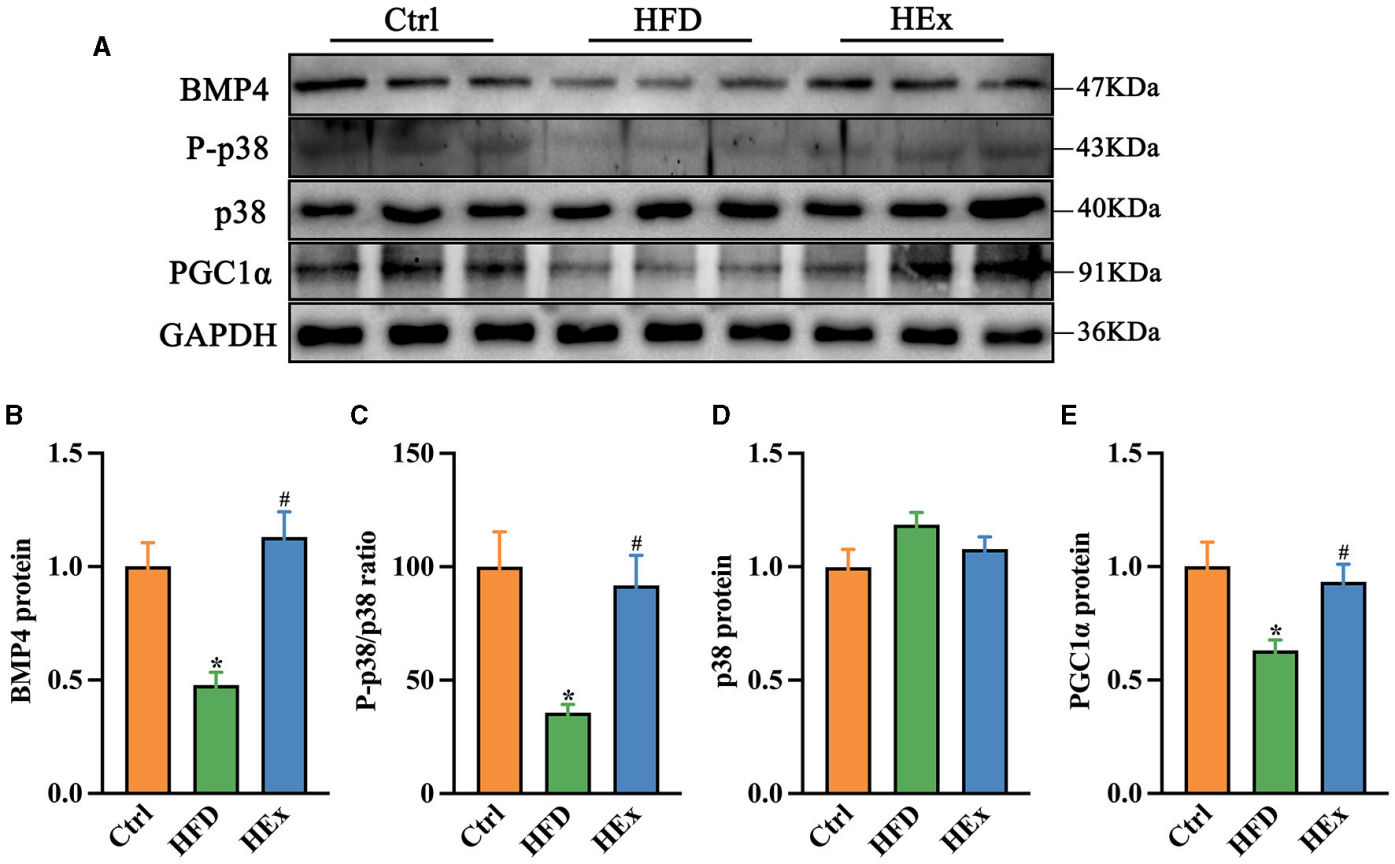
Effects of aerobic exercise on the BMP4 signaling pathway in PVAT of rats fed with a high-fat diet. **(A)** Representative Western blot images of BMP4, P-p38, p38, PGC1α, and internal control GAPDH in PVAT. **(B–E)** Protein levels of BMP4, P-p38/p38, p38 and PGC1α (*n* = 6). The data are presented as mean ± SEM. The results are significant compared to the control group (*p < 0.05) and the HFD group (^#^*p* < 0.05).

Consistent with gene expression data, Western blot results showed decreased protein levels of BMP4 and PGC1α along with a decreased ratio of P-p38/p38MAPK in PVAT of obese rats. However, the loss of these protein levels was significantly restored after exercise training (*p* < 0.05). The quantified protein levels of BMP4 and PGC1α and the ratio of P-p38/p38/MAPK in HEx group were almost similar to the control group (Figures 7A–E). These results provide convincing evidence that aerobic exercise can promote both transcriptional activation and protein levels of BMP4 and associated signaling molecules in PVAT, thereby regulating the phenotypic transformation of adipose tissue in obese rats.

## 4 Discussion

For the first time, our findings demonstrated that aerobic exercise training can ameliorate the high-fat diet-induced thoracic PVAT dysfunction, possibly by promoting the BMP4-mediated adipose tissue browning in obese rats. Chronic HFD-induced CVD risk factors, represented by a significant weight gain and dyslipidemia, were effectively mitigated by aerobic exercise. The impaired secretion of leptin and elevated release of IL-1β concentration with HFD were reversed by exercise. In addition, exercise training ameliorated HFD-induced white adiposity, corrected morphological abnormalities, and reduced the lipid droplet size in PVAT. Furthermore, the exercise intervention attenuated the whitening of PVAT via restoration of UCP1 and suppression of FABP4 proteins against HFD. This beneficial effect of exercise was accompanied by balancing the inflammatory response in PVAT of obese rats. Our findings further witnessed that exercise promoted transcriptional activation and protein levels of BMP4 and associated signaling molecules in PVAT. These findings indicate that exercise training can remodel the adipocyte phenotypic transformation of thoracic PVAT in obese rats possibly by promoting the inflammatory response and BMP4-mediated signaling cascades.

One of our convincing findings is that aerobic exercise training substantially decreased HFD-induced weight gain or obesity in rats. This anti-obesity effect of exercise was accompanied by a notable decrease of circulating TC, TG, and LDL concentrations. These findings were consistent with previous studies, in which exercise attenuated HFD-induced obesity and dyslipidemia in rats (32, 33). Dyslipidemia, characterized by an abnormal elevation of TC, TG, and LDL concentrations, is a hallmark of incidence of CVDs or atherosclerosis (34). Excessive accumulation of triglycerides in PVAT leads to enlarge adipocytes due to excess lipid droplets. WAT is responsible for the storage of excessive energy in the form of lipids (large single droplets), while BAT stores large amounts of lipids due to its thermogenic property (contains multiple smaller lipid droplets) (10, 21). In our study, HFD not only elevated the circulating lipid concentrations but also increased the average size of lipid droplets in PVAT. The enlarged lipid droplet size, morphological alterations, and architectural derangements of PVAT in obese rats demonstrate that the thoracic aorta is predominantly surrounded by the WAT. Such increased accumulation of lipids on PVAT results in altering the perivascular adiposity and vasoconstriction over a period of time (2, 35). We then found that the PVAT-regulated vasodilatory response was decreased with HFD, which indicates dysfunctional PVAT due to adiposity.

It is worth to note that exercise decreased the circulating lipid concentrations and lipid droplet size on PVAT. The smaller lipid droplets in the HEx group is a sign of BAT-like characteristics and normalized PVAT morphological alterations. This antilipidemic effect of exercise further contributed to attenuate the anti-contractile response of aortic rings and improved the vascular reactivity in obese rats. A study by Araujo et al. has shown that exercise training ameliorates the anti-contractile function of PVAT, possibly by a greater reduction of PVAT mass and diminution of morphological alterations in aortic rings of HFD rats (9). Exercise training can reverse the HFD-induced abrupt changes in PVAT adiposity and contractile vascular reactivity in obese mice, and these changes were represented by a mix of BAT and WAT with moderate architectural alterations in PVAT (35). Furthermore, exercise training can reduce PVAT amount and morphological alterations without affecting the anti-contractile activity of PVAT and PVAT-derived leptin, adiponectin and TNF-α levels in healthy rats (36). These findings demonstrate that exercise can restore the PVAT function, which could contribute to decrease the risk of developing CVDs against HFD.

The beneficial effects of exercise are possibly associated with improved inflammatory response and/or tight control of molecular events in adipose tissue transformation in obese rats. To elucidate this phenomenon, changes in pro- and anti-inflammatory biomarkers in response to HFD and exercise were determined. HFD impairs circulating adipokines (leptin and adiponectin) and induces a pro-inflammatory response. Studies have shown that obesity can impair adipose tissue function and promote adipokine secretion, thereby causing inflammation (37). Augmented leptin and decreased adiponectin concentrations in blood and PVAT demonstrate the malfunctioning of adipose tissue and inflammatory status in obese rats. This phenomenon was further supported by increased MCP-1 and decreased IL-10 in PVAT of obese rats. Chronic inflammation lead to cellular and tissue damage, and it plays a critical role in the pathophysiology of vascular dysfunction (38). In conditions of obesity, adipose tissue undergoes a profound pathological change in its morphology, size, and functional efficiency (39). HFD-induced inflammation in our study is a scenario that explains whitening of adipose tissue (conversion of BAT to WAT) with decreased BAT and increased WAT contents.

In our study, exercise training attenuated HFD-induced inflammation by restoring the anti-inflammatory adiponectin and IL-10 mRNA as well as inhibiting the pro-inflammatory cytokines (leptin, IL-1β, and MCP-1). Exercise produces some beneficial adaptations to WAT; however, the responses of inflammatory biomarkers are equivocal. For instance, aerobic exercise decreased periaortic adipose tissue without altering the PVAT-derived leptin, adiponectin, and TNF-α in healthy rats (36). In another study, exercise training decreased the PVAT mass, but it did not diminish the MCP-1 elevation in PVAT of obese mice (40). On the other hand, increased PVAT mass in obesity is accompanied by an increased macrophage infiltration, elevated MCP-1, and decreased adiponectin secretion (41). Therefore, exercise-induced reduction of MCP-1 expression and restoration of adiponectin (circulation and tissue) in our study demonstrate the shifting of pro-inflammatory status to anti-inflammatory status. Furthermore, PVAT-derived adiponectin acts as a physiological modulator of vascular tonus by inhibiting the pro-inflammatory cytokine secretion through the nuclear factor-κB (NF-κB)-dependent pathway (36, 42). Since inflammation and oxidative stress are critically involved in PVAT dysfunction (24), the improved anti-inflammatory status, dyslipidemia, and reduced lipid droplet size with exercise might have contributed to attenuate HFD-induced PVAT dysfunction, possibly by modulating the key molecular events in PVAT phenotype shift.

Several studies have documented that exercise-induced restoration of PVAT function is mediated by decreasing obesity or controlling adipocyte phenotype transformation (24, 35). Nevertheless, the key factors involved in adipocyte phenotype shift during exercise, particularly in diet-induced obese rats remain unexplored. For the first time, we demonstrated that exercise training attenuates HFD-induced whitening while promoting browning of adipose tissue in obese rats. This was evidenced by restored UCP1 and downregulated FABP4 protein levels in PVAT of obese exercised rats. UCP1 is a hallmark protein in BAT, responsible for diet-induced thermogenesis and playing a role in controlling obesity (43). FABP4 is highly expressed in adipocytes and is involved in obesity-related metabolic diseases, including diabetes and atherosclerosis (44). Obesity is associated with a loss of UCP1 (brown-like phenotype marker) and/or elevation of FABP4 (white-like phenotype marker) (44, 45). In agreement with previous reports, our findings witnessed a notable loss of UCP1 and gain of FABP4 expressions in PVAT of obese rats. Since UCP1 loss and increased inflammatory status are associated with whitening of adipose tissue (46), exercise-induced restoration of UCP1 expression and inhibition of inflammation contribute to promote adipocyte phenotype shift or the browning process. Exercise training upregulated UCP1 protein content in WAT of mice, and PGC-1α is required for such elevation (47). Increased UCP1 protein expression in skeletal muscle after exercise also suggesting phenotype switch to beige/brown muscle lipid in HFD-fed mice (48). It is also claimed that pharmacological modification of FABP4 function by specific inhibitors would be a novel therapeutic strategy to treat CVD, obesity and atherosclerosis (49). Similar to BAT-like characteristics, exercise suppressed WAT-like characteristics of FABP4 expression in PVAT, thereby reversing adipocyte phenotype shift and developing atherosclerosis in obese rats.

The activation of adipose tissue browning and thermogenesis provide a new strategy to counter the obesity-mediated metabolic diseases. PDGFRα cells are bipotential adipocyte progenitors and can differentiate into either white or beige adipocytes (12). Double immunofluorescence staining of PDGFRα and Ki67 showed that mast cell inactivation enhanced the proliferation of ki67^+^PDGFRα^+^ adipose progenitor cells, thereby promoting beige fat biogenesis in subcutaneous adipose tissue (49). Beige adipocyte progenitors expressing PDGFRα were significantly reduced in WAT following HFD feeding. Therefore, it is possible that the reduction in the numbers of PDGFRα expressing progenitors is an underlying mechanism for the attenuation of beige adipocyte development in obese mice (50). Aerobic exercise can ameliorate HFD-induced metabolic disorders and vascular dysfunction and increase adipose progenitor cell population in brown adipose tissue, which may contribute to enhance fictional brown adipose tissue (51). Interestingly, our findings revealed no change in PDGFRα and ki67 expression in PVAT. This discrepancy could be attributed to the heterogeneity of PVAT, which originates from multiple adipose progenitor cells. Ye et al. (52) reported that PVAT in the anterior thoracic aorta were developed from SM22α^+^ progenitor cells, while adipocytes in the lateral thoracic aorta were developed from SM22α^+^ and Myf5^+^ cells. Given that the effects of aerobic exercise on other distinct adipose fat precursor cells were not assessed in the present study, future research should consider exploring on this aspect.

It has been proven that BMP4 regulates white adipogenesis by transforming the brown-to-white shift. The absence of BMP4 in PVAT reduces BAT-characteristic gene expressions, increases pro-inflammatory mediators, and exacerbates atherosclerosis plaque formation, while activation of BMP4 signaling reverses these pathological features in ApoE^−/−^ mice (18). Chronic HFD feeding in our study caused a significant reduction of BMP4 mRNA and protein levels in PVAT suggesting the diet-induced whitening of thoracic PVAT. However, transcriptional activation and increased amounts of BMP4 by exercise training promote PVAT browning, which is also supported by decreased pro-inflammatory mediators, smaller lipid droplets, and restored UCP1 expression in obese rats. A study by Qian and colleagues demonstrated that overexpression of BMP4 in WAT resulted in a reduction of adipocyte size, WAT mass, and fat pad size. This also resulted in an increase in the number of white adipocyte cell types with brown adipocyte characteristics in mice (15). Increased circulating BMP4 in mice has been reported to target subcutaneous WAT, promoting its browning by augmented UCP1, increased energy expenditure, and enhanced mitochondrial activity (17). At the molecular level, BMP4 regulate adipogenic function via two signaling cascades, the p38/MAPK and Smad pathways (53). The p38 pathway mainly controls the activation of BAT and browning of WAT by inducing UCP1 transcription through the activation of cAMP response element-binding protein (CREB), ATF2, and PGC1α (54). Chronic HFD feeding inhibits activation of the p38/MAPK signaling pathway, whereas aerobic exercise training significantly improves p38 phosphorylation levels and lipid metabolism in obese mice (55). Similar to this result, our findings also demonstrated restoration of the P-p38/p38MAPK ratio against HFD-mediated loss in PVAT. It has been shown that PGC1α acts as a key regulator that is transactivated by ATF2 mainly through the BMP4–p38/MAPK signaling pathway (15). In our study, chronic HFD feeding in rats led to a significant reduction of BMP4-mediated signaling molecules, p38/MAPK, ATF2, PGC1α, and Smads in PVAT. A recent study reported that browning of adipose tissue was induced by the overexpression of BMP4 in WAT via the activation of Smad1/5/8 and p38/MAPK pathways (15). It is worthy to note that exercise training promoted the transcriptional activation of these signaling molecules (p38/MAPK, ATF2, PGC1α, and Smad5) along with BMP4 in obese rats. Consistent with mRNA expressions, the protein levels of PGC1α and p38/MAPK were also elevated in obese exercised rats. Laboratory evidence revealed that activation of the BMP4-mediated p38/MAPK/ATF2 pathway and PGC1α expression play key role in induction of WAT into BAT-like tissue (15). Furthermore, exercise training upregulated PGC1α and UCP7 protein contents in epididymal WAT and contributed to brown adipose-like phenotype modulation in HFD fed rats (56). Finally, our findings suggest that aerobic exercise can modulate the protein and gene levels of BMP4 downstream signaling pathways that are associated with PVAT phenotypic shift in obese rats.

## 5 Conclusion

This study highlighted that 6-week aerobic exercise can decrease bodyweight and attenuate dyslipidemia in rats fed with HFD. The beneficial effect of exercise against HFD is accompanied by the improved inflammatory response and decreased lipid deposition in thoracic PVAT. Exercise training promotes browning of adipose tissue and restores the vascular function. Our findings suggest that regular exercise training is an effective strategy to ameliorate HFD-induced PVAT dysfunction through improved inflammatory response and BMP4-mediated signaling pathways.

## Data availability statement

The original contributions presented in the study are included in the article/Supplementary material, further inquiries can be directed to the corresponding authors.

## Ethics statement

The entire study design and protocols were reviewed and approved by the Zhejiang Normal University, Animal Ethical Committee with the reference number ZSDW2022027. The study was conducted in accordance with the local legislation and institutional requirements.

## Author contributions

XL: Data curation, Formal analysis, Investigation, Methodology, Writing—original draft. XJ: Data curation, Formal analysis, Investigation, Methodology, Resources, Writing—original draft. JH: Data curation, Formal analysis, Investigation, Methodology, Resources, Writing—original draft. MD: Formal analysis, Investigation, Methodology, Writing—original draft. SL: Conceptualization, Resources, Validation, Visualization, Writing—review & editing. MK: Conceptualization, Data curation, Supervision, Validation, Visualization, Writing—review & editing. YQ: Project administration, Resources, Software, Writing—original draft. TL: Data curation, Methodology, Resources, Software, Writing—original draft. LW: Investigation, Project administration, Resources, Supervision, Validation, Visualization, Writing—review & editing. WL: Conceptualization, Data curation, Funding acquisition, Investigation, Project administration, Supervision, Validation, Visualization, Writing—original draft.
