# Supplementary material for: Exercise attenuates high-fat diet-induced PVAT dysfunction through improved inflammatory response and BMP4-regulated adipose tissue browning
Source: Front Nutr. 2024 May 9;11:1393343. doi: 10.3389/fnut.2024.1393343 (PMC11111863; doi:10.3389/fnut.2024.1393343)
Supplement: Supplementary file 1 [file Image_1.pdf]

Supplementary Material

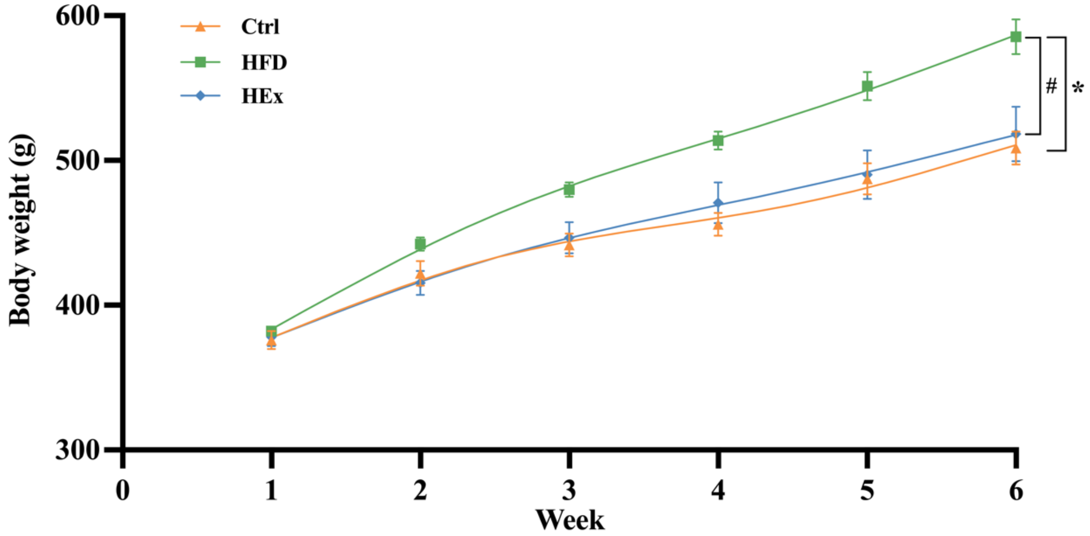

Supplementary Figure 1. The effects of aerobic exercise on body weight gain in rats fed a high-fat diet (n = 6). Data are presented as mean ± SEM. Results are significant compared with control $^{*}p < 0.05$; compared with HFD $^{\#}p < 0.05$.
